# Image-Guided Cancer Nanomedicine

**DOI:** 10.3390/jimaging4010018

**Published:** 2018-01-11

**Authors:** Dong-Hyun Kim

**Affiliations:** 1 Department of Radiology, Feinberg School of Medicine, Northwestern University, Chicago, IL 60611, USA; 2 Robert H. Lurie Comprehensive Cancer Center, Chicago, IL 60611, USA

**Keywords:** nanomedicine, image, image-guided nanomedicine, nanoparticles, cancer

## Abstract

Multifunctional nanoparticles with superior imaging properties and therapeutic effects have been extensively developed for the nanomedicine. However, tumor-intrinsic barriers and tumor heterogeneity have resulted in low in vivo therapeutic efficacy. The poor in vivo targeting efficiency in passive and active targeting of nanotherapeutics along with the toxicity of nanoparticles has been a major problem in nanomedicine. Recently, image-guided nanomedicine, which can deliver nanoparticles locally using non-invasive imaging and interventional oncology techniques, has been paid attention as a new opportunity of nanomedicine. This short review will discuss the existing challenges in nanomedicine and describe the prospects for future image-guided nanomedicine.

## 1. Nanomedicine

Various nanomaterials, having special functions that have not been observed in bulk materials, can provide opportunities for innovative biomedical applications. Nanomedicine has been one of the key research areas among those various applications of nanotechnology for about 20 years. Cancer is the 2nd most common cause of death and cancer cases keep rising in every year [1]. Conventional therapies have not shown any significant progress or outcomes for treating cancers. Disruptive innovations are desperately needed to more effectively treat patients with cancer. Cancer nanomedicine using unique features of nanomaterials has been expected to provide new opportunities in early diagnosis, imaging and treatment of cancers. The small size, high surface area, aqueous solubility, and multi-functionality of nanoparticles have created new biomedical applications. Indeed, the novel properties of nanoparticles have demonstrated the ability to interact with complex cellular functions in new ways. This rapidly growing field as an inter-disciplinary research develops multifunctional nanostructures and approaches that can target, diagnose, and treat devastating cancers. With extensive efforts, liposomes and lipid-based nanoparticles have been FDA approved to deliver and enhance the bioavailability of doxorubicin and other drugs [2,3]. Micelles and nanocomplexes has also improved the pharmacokinetics (PK) and biodistribution of hydrophobic drug molecules [4]. In addition, carboxymethyldextran-coated iron oxide nanoparticles have been approved for iron supplements in drugs and are now being tested for MR contrast agents in clinics [5–7]. Approximately, 100 nanomedicine products have been commercialized and marketed [2]. Other various nanomaterials are on about 800 clinical trials [8].

In preclinical tests, numerous nanomaterials demonstrate very promising properties for cancer imaging and therapeutics. However, only a few nanomaterials composed of Fe, Si, Au, polysaccharides polymers or natural products have been considered for potential clinical applications. Representatively, iron oxide nanoparticles, which are one of the elements in blood, have been used for cellular hyperthermia and MR imaging contrast [9,10]. The superparamagnetic properties of nanoscale iron oxide particles have been using for those applications and beyond. The magnetic properties and functions for medicine are readily tailored for each purpose by changing the size and structure of the nanoparticles. Recently, anisotropic and high-complexity Au nanostructures such as hyper-branched or dendritic structures also have been observed to be advantageous, because they provide a larger number of available active sites and surface atoms per unit area compared to spherical nanoparticles [11]. Various shaped metallic nanoparticles having specific light absorption properties generate robust heating for the local ablation therapies. High-density metallic nanoparticles allow a CT imaging contrast effect [12]. Disk-shaped Au-coated magnetic particles with a magnetic spin vortex can directly kill cancer cells with magneto-mechanical stimulations modulated by an external magnetic field [13]. Temperature-sensitive polymeric micelles effectively deliver drug molecules at a specific temperature [14]. Magnetic clusters enhance the MR imaging properties and at the same time carry much of drug with the nanopores [15]. Further mesoporous silica nanoparticles have shown great potential for the drug carriers [16,17]. Upconversion nanoparticles have been developed for stable luminescent and multimodal imaging functions in pre-/intra-/post-operative imaging [18]. Those proposed nanomedicines using novel nanoparticles should be a desirable new approach to treat cancers.

## 2. Current Limitations of Nanomedicine

However, big challenges of those nanoparticles for nanomedicine applications has been issued during recent in vivo and clinical translations [19]. In 2016, Wilhelm et al. [20] reviewed the literature from the past 10 years on nanoparticles-based nanomedicine; they reported that only 0.7% [median] of the administered nanoparticle dose was delivered to a solid tumor. The enhanced permeability and retention (EPR) effect and active targeting using tumor specific molecules are regarded as promising approaches for the tumor targeting, but RES sequestration, tumor-intrinsic barriers and tumor heterogeneity resulted in extremely low tumor targeting and tumor uptake efficiency [20–22]. This low targeting efficiency negatively affects the translation of nanomedicine to clinical applications. Hence, current future cancer nanomedicine strongly requires more localized and personalized approaches considering the tumor heterogeneity. More efforts for in-depth understanding of nanoparticles and tumor interactions are needed [2]. Eventually, nanomedicine approaches should be tailored and personalized based on medical diagnosis and imaging. Medical image-guided interventional oncology approaches should be one of the promising solutions for current nanomedicine.

## 3. Image Guided Cancer Nanomedicine: A New Opportunity

Interventional oncology is a subspecialty field of interventional radiology that performs the diagnosis and treatment of cancer using targeted minimally invasive procedures performed under image guidance. It employs X-ray, ultrasound, computed tomography (CT) or magnetic resonance imaging (MRI) to help guide miniaturized instruments (e.g., intravascular catheter, biopsy needles, ablation electrodes) to allow targeted and precise treatment of solid tumors located in various organs of the human body. Advances in medical imaging and image guidance for the detection, characterization, targeting and therapy of cancers now allow for minimally invasive image-guided treatment of many solid tumors without the toxicity of chemotherapy and radiation. The image-guided procedures have been shown to result in fewer complications, faster recoveries, and reduced costs [23–25]. The most widely practiced procedures are transcatheter-directed therapies with intra-tumoral or intra-arterial delivery and percutaneous or endoscopic ablative therapies. Transcatheter-directed therapies such as transcatheter arterial embolization (TAE) and chemoembolization (TACE) are catheter-based intra-arterially delivered tumor treatments. Ablative therapies such as radiofrequency ablation (RFA) and cryo-ablation generally involve the destruction of the lesion via a percutaneously placed needle. Medical imaging plays key roles in those image-guided therapies and interventional procedures. Those roles are (a) preprocedure planning (identifying tumor volume); (b) intraprocedural targeting (guiding catheter delivery); (c) intraprocedural monitoring (monitoring tumor tissue changes caused by the treatment during the procedure); (d) intraprocedural control (making adjustments); and (e) postprocedure assessment (measuring effectiveness and for further intervention). Contrast agents are often required to highlight a target site that is not visualized well on unenhanced scans in pre-, intra- and post-procedural therapies.

**Figure 1:**
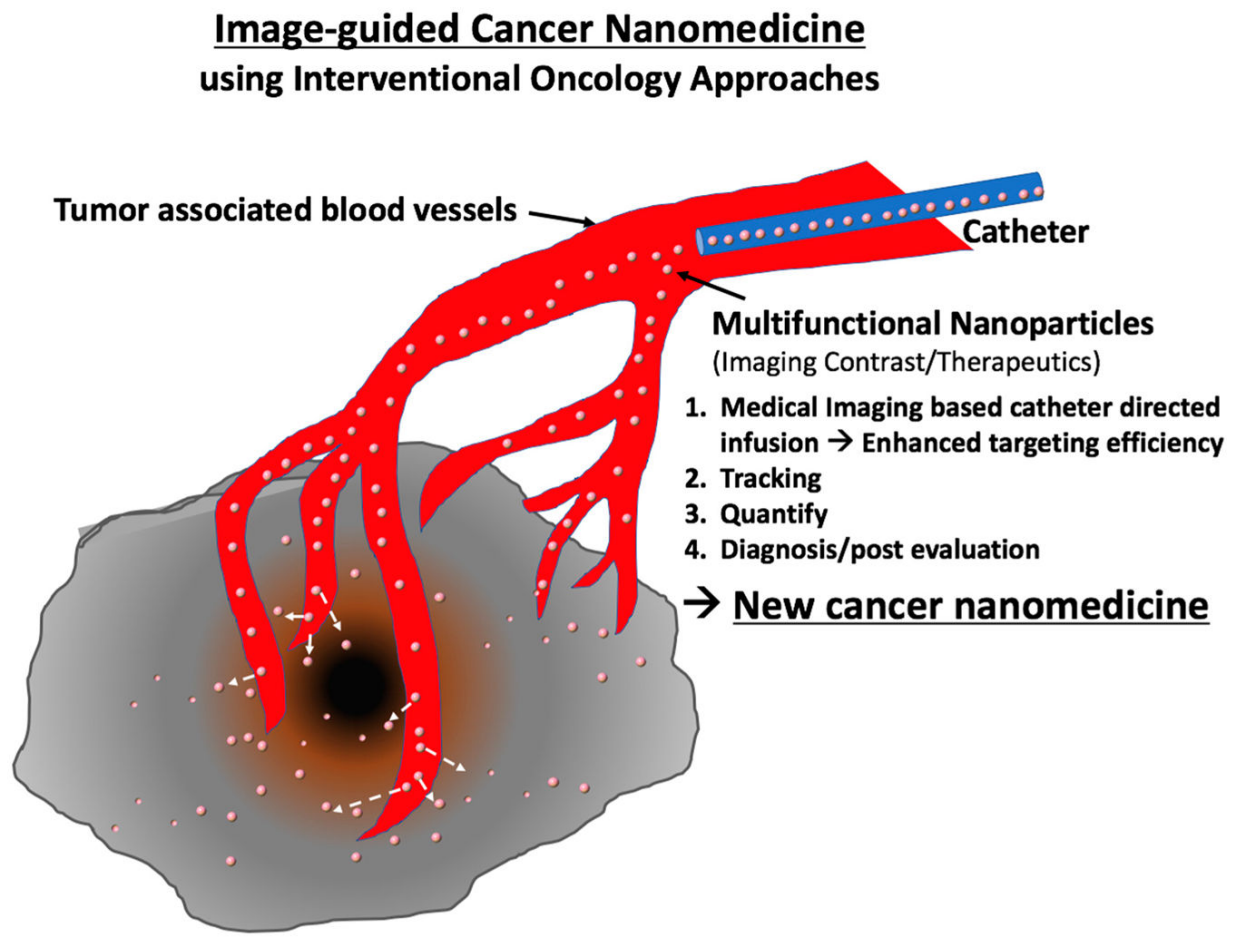
Image-guided Cancer Nanomedicine. Image-guided infusion of nanomedicine using interventional procedures allows personalized therapeutics with highly localized nanotherapeutics.

Although those interventional approaches mainly have been used for traditional local therapies such as radiofrequency/cryo/chemical tumor ablation, focal laser ablation, tumor (chemo-) embolization, local drug infusion and so on, those conventional interventional therapeutics are conveniently combined with multifunctional nanoparticle-based nanomedicine. Recently, various image-guided cancer nanomedicine approaches have been tested and have shown promising results in preclinical settings (Table 1). Advanced functions of nanoparticles provide high imaging contrast effects during image-guided therapeutic procedures and more tumor-specific triggered therapeutics at the same time. These features also suggest a new opportunity of nanomedicine that has been stagnant with low tumor targeting and toxicity issues for clinical translation. Now, emerging next-generation nanomedicine—“image-guided cancer nanomedicine”—combined with interventional oncology approaches fulfills minimal systemic distribution, homogeneous distribution at targeted sites and high local delivery of nanomedicine resulting in enhancing the efficacy of cutting-edge nanomedicines (Figure 1). Furthermore, the image-guided delivery of nanomedicine will be important in future clinical practice (Figure 1). First, an effective dosage of nanomedicine can be highly localized in tumor regions with minimized systemic toxicity; second, it is possible to monitor and confirm whether the nanoparticles-based nanomedicine is properly delivered to the disease site after injection (local infusion and tracking); third, an amount of the injected nanoparticles can be quantitatively analyzed to determine the amount of the post infusion (non-invasive quantification); finally, distribution of the injected nanoparticles in the body can be monitored for a long-term period (diagnosis and post evaluation). The proposed new image-guided cancer nanomedicine approaches should eventually permit patient-specific dosimetry and tumor-specific treatment of cancers for the superior therapeutic effect in a personalized manner [18,26–28]. At the same time, it is expected that the use of nanomedicine techniques in interventional oncology will open a new chapter for exceptional therapeutic efficacies [15,29]. It is worth noting that image-guided cancer nanomedicine incorporates new medical imaging techniques, nanoparticles, molecular entities and novel classes of therapeutic agents (siRNA, mRNA, gene editing, immune checkpoint inhibitors) as well as existing drugs/therapeutics. Strong collaborative multidisciplinary teams including clinicians, basic scientists and nano-scientists are essential for advancing the image-guided cancer nanomedicine for clinical translation.

## Figures and Tables

**Table 1:** Recent Image-guided Cancer Nanomedicine Approaches

Therapeutics	Cancer	Imaging	Nanoparticles	References
Image-guided Delivery	Brain cancer [30] Prostate cancer [31]	MRI/CT [30] MRI/fluorescent [31]	Hybrid iron oxide/gold [30] Silica/melanin nanoparticles [31]	[30,31]
Image-guided radiation	Lung carcinoma [32]	MRI/CT [32]	Bismuth/gadolinium [32]	[32]
Image-guided drug delivery	peritoneal tumors [33] pancreatic tumors [16,34] Hepatocellular carcinoma [35,36]	MRI [33] MRI [16,34,36] MRI/CT [35]	Iron oxide nanoparticles [33] Iron oxide nanoparticles [16,34,36] Iron oxide/gold nanoparticles [35]	[16,33–36]
Image-guided surgery	Breast cancer and hepatocellular carcinoma [37] Liver cancer [18]	Radiofluorescent [37] MRI/Luminescent [18]	Europium oxide nanoparticle [37] Upconversion nanoparticles [18]	[18,37]
Image-guided photodynamic therapy	Ovarian cancer [38]	Near-infrared fluorescence imaging (NIRFI), MRI, PET [38]	Nanoporphyrin [38]	[38]
Image-guided photothermal therapy	Pancreatic cancer [39] Prostate cancer [40] Colorectal cancer [41]	fluorescent [39] MRI [40] MRI [41]	Branched gold nanoparticles [39] Gold nanoparticles [40] Hybrid gold/iron oxide nanoparticles [41]	[39–41]
Image-guided immunotherapy	Liver cancer [42]	MRI [42]	Iron oxide nanocubes [42]	[42]

## References

[1] American Cancer Society (2017). Cancer Facts & Figures.

[2] Chen H, Zhang W, Zhu G, Xie J, Chen X (2017). Rethinking cancer nanotheranostics. Nat Rev Mater.

[3] Shi J, Kantoff PW, Wooster R, Farokhzad OC (2017). Cancer nanomedicine: Progress, challenges and opportunities. Nat Rev Cancer.

[4] Kim BY, Rutka JT, Chan WC (2010). Nanomedicine. N Engl J Med.

[5] Thu MS, Bryant LH, Coppola T, Jordan EK, Budde MD, Lewis BK, Chaudhry A, Ren J, Varma NR, Arbab AS (2012). Self-assembling nanocomplexes by combining ferumoxytol, heparin and protamine for cell tracking by magnetic resonance imaging. Nat Med.

[6] Weissleder R, Nahrendorf M, Pittet MJ (2014). Imaging macrophages with nanoparticles. Nat Mater.

[7] Khurana A, Nejadnik H, Gawande R, Lin GT, Lee S, Messing S, Castaneda R, Derugin N, Pisani L, Lue TF (2012). Intravenous Ferumoxytol Allows Noninvasive MR Imaging Monitoring of Macrophage Migration into Stem Cell Transplants. Radiology.

[8] Bobo D, Robinson KJ, Islam J, Thurecht KJ, Corrie SR (2016). Nanoparticle-Based Medicines: A Review of FDA-Approved Materials and Clinical Trials to Date. Pharm Res.

[9] Kim DH, Nikles DE, Johnson DT, Brazel CS (2008). Heat generation of aqueously dispersed CoFe_2_O_4_ nanoparticles as heating agents for magnetically activated drug delivery and hyperthermia. J Magn Magn Mater.

[10] Kim DH, Nikles DE, Brazel CS (2010). Synthesis and Characterization of Multifunctional Chitosan-MnFe2O4 Nanoparticles for Magnetic Hyperthermia and Drug Delivery. Materials.

[11] Hao F, Nehl CL, Hafner JH, Nordlander P (2007). Plasmon resonances of a gold nanostar. Nano Lett.

[12] Park W, Cho S, Huang X, Larson AC, Kim DH (2017). Branched Gold Nanoparticle Coating of Clostridium novyi-NT Spores for CT-Guided Intratumoral Injection. Small.

[13] Kim DH, Rozhkova EA, Ulasov IV, Bader SD, Rajh T, Lesniak MS, Novosad V (2010). Biofunctionalized magnetic-vortex microdiscs for targeted cancer-cell destruction. Nat Mater.

[14] Kim DH, Vitol EA, Liu J, Balasubramanian S, Gosztola DJ, Cohen EE, Novosad V, Rozhkova EA (2013). Stimuli-Responsive Magnetic Nanomicelles as Multifunctional Heat and Cargo Delivery Vehicles. Langmuir.

[15] Jeon MJ, Gordon AC, Larson AC, Chung JW, Kim YI, Kim DH (2016). Transcatheter intra-arterial infusion of doxorubicin loaded porous magnetic nano-clusters with iodinated oil for the treatment of liver cancer. Biomaterials.

[16] Kim DH, Guo Y, Zhang Z, Procissi D, Nicolai J, Omary RA, Larson AC (2014). Temperature-sensitive magnetic drug carriers for concurrent gemcitabine chemohyperthermia. Adv Healthc Mater.

[17] Sun J, Kim DH, Guo Y, Teng ZG, Li YJ, Zheng LF, Zhang ZL, Larson AC, Lu GM (2015). A c(RGDfE) conjugated multi-functional nanomedicine delivery system for targeted pancreatic cancer therapy. J Mater Chem B.

[18] Lee J, Gordon AC, Kim H, Park W, Cho S, Lee B, Larson AC, Rozhkova EA, Kim DH (2016). Targeted multimodal nano-reporters for pre-procedural MRI and intra-operative image-guidance. Biomaterials.

[19] Nanotechnology Fact Sheet.

[20] Wilhelm S, Tavares AJ, Dai Q, Ohta S, Audet J, Dvorak HF, Chan WCW (2016). Analysis of nanoparticle delivery to tumours. Nat Rev Mater.

[21] McNeil SE (2016). Evaluation of nanomedicines: Stick to the basics. Nat Rev Mater.

[22] Wilhelm S, Tavares AJ, Chan WCW (2016). Reply to “Evaluation of nanomedicines: Stick to the basics”. Nat Rev Mater.

[23] Silverman SG, Deuson TE, Kane N, Adams DF, Seltzer SE, Phillips MD, Khorasani R, Zinner MJ, Holman BL (1998). Percutaneous abdominal biopsy: Cost-identification analysis. Radiology.

[24] Link RE, Permpongkosol S, Gupta A, Jarrett TW, Solomon SB, Kavoussi LR (2006). Cost analysis of open, laparoscopic, and percutaneous treatment options for nephron-sparing surgery. J Endourol.

[25] Solomon SB, Silverman SG (2010). Imaging in interventional oncology. Radiology.

[26] Kim DH, Chen J, Omary RA, Larson AC (2015). MRI visible drug eluting magnetic microspheres for transcatheter intra-arterial delivery to liver tumors. Theranostics.

[27] Kim DH, Choy T, Huang S, Green RM, Omary RA, Larson AC (2014). Microfluidic fabrication of 6-methoxyethylamino numonafide-eluting magnetic microspheres. Acta Biomater.

[28] Park W, Cho S, Han J, Shin H, Na K, Lee B, Kim DH (2018). Advanced smart-photosensitizers for more effective cancer treatment. Biomater Sci.

[29] Chen J, White SB, Harris KR, Li W, Yap JW, Kim DH, Lewandowski RJ, Shea LD, Larson AC (2015). Poly(lactide-*co*-glycolide) microspheres for MRI-monitored delivery of sorafenib in a rabbit VX2 model. Biomaterials.

[30] Tomitaka A, Arami H, Raymond A, Yndart A, Kaushik A, Jayant RD, Takemura Y, Cai Y, Toborek M, Nair M (2017). Development of magneto-plasmonic nanoparticles for multimodal image-guided therapy to the brain. Nanoscale.

[31] Cho S, Park W, Kim DH (2017). Silica-Coated Metal Chelating-Melanin Nanoparticles as a Dual-Modal Contrast Enhancement Imaging and Therapeutic Agent. ACS Appl Mater Interfaces.

[32] Detappe A, Thomas E, Tibbitt MW, Kunjachan S, Zavidij O, Parnandi N, Reznichenko E, Lux F, Tillement O, Berbeco R (2017). Ultrasmall Silica-Based Bismuth Gadolinium Nanoparticles for Dual Magnetic Resonance-Computed Tomography Image Guided Radiation Therapy. Nano Lett.

[33] Gao N, Bozeman EN, Qian W, Wang L, Chen H, Lipowska M, Staley CA, Wang YA, Mao H, Yang L (2017). Tumor Penetrating Theranostic Nanoparticles for Enhancement of Targeted and Image-guided Drug Delivery into Peritoneal Tumors following Intraperitoneal Delivery. Theranostics.

[34] Zhou H, Qian W, Uckun FM, Wang L, Wang YA, Chen H, Kooby D, Yu Q, Lipowska M, Staley CA (2015). IGF1 Receptor Targeted Theranostic Nanoparticles for Targeted and Image-Guided Therapy of Pancreatic Cancer. ACS Nano.

[35] Kim D-H, Li W, Chen J, Zhang Z, Green RM, Huang S, Larson A (2016). Multimodal Imaging of Nanocomposite Microspheres for Transcatheter Intra-Arterial Drug Delivery to Liver Tumors. Sci Rep.

[36] Park W, Chen J, Cho S, Park SJ, Larson AC, Na K, Kim DH (2016). Acidic pH-Triggered Drug-Eluting Nanocomposites for Magnetic Resonance Imaging-Monitored Intra-arterial Drug Delivery to Hepatocellular Carcinoma. ACS Appl Mater Interfaces.

[37] Hu Z, Chi C, Liu M, Guo H, Zhang Z, Zeng C, Ye J, Wang J, Tian J, Yang W (2017). Nanoparticle-mediated radiopharmaceutical-excited fluorescence molecular imaging allows precise image-guided tumor-removal surgery. Nanomedicine.

[38] Li Y, Lin TY, Luo Y, Liu Q, Xiao W, Guo W, Lac D, Zhang H, Feng C, Wachsmann-Hogiu S (2014). A smart and versatile theranostic nanomedicine platform based on nanoporphyrin. Nat Commun.

[39] Kim DH, Larson AC (2015). Deoxycholate bile acid directed synthesis of branched Au nanostructures for near infrared photothermal ablation. Biomaterials.

[40] Zhao K, Cho S, Procissi D, Larson AC, Kim DH (2017). Non-invasive monitoring of branched Au nanoparticle-mediated photothermal ablation. J Biomed Mater Res B Appl Biomater.

[41] White SB, Kim DH, Guo Y, Li W, Yang Y, Chen J, Gogineni VR, Larson AC (2017). Biofunctionalized Hybrid Magnetic Gold Nanoparticles as Catalysts for Photothermal Ablation of Colorectal Liver Metastases. Radiology.

[42] Park W, Gordon AC, Cho S, Huang X, Harris KR, Larson AC, Kim DH (2017). Immunomodulatory Magnetic Microspheres for Augmenting Tumor-Specific Infiltration of Natural Killer (NK) Cells. ACS Appl Mater Interfaces.

